# The influence of social media and video-on-demand platforms on the appeal and consumption of tobacco and emerging tobacco products: A cross-sectional study

**DOI:** 10.18332/tid/194491

**Published:** 2024-10-25

**Authors:** Diego de Haro, Maria Luz Amador, Mario Lopez-Salas, Karen L. Ramirez Cervantes, Antonio Yanes-Roldan, Belén Fernández, Jose M. Martin-Moreno

**Affiliations:** 1 Spanish Association Against Cancer, Madrid, Spain; 2 Department of Preventive Medicine and Public Health, Instituto de Investigación Sanitaria, University of Valencia, Valencia, Spain

**Keywords:** tobacco advertising, youth, prevention, tobacco use, electronic cigarettes

## Abstract

**INTRODUCTION:**

Digital platforms serve as an avenue for the tobacco industry to promote both conventional tobacco and emerging products, with a notable focus on capturing the attention of young people through sophisticated marketing campaigns. This research aims to analyze the prevalence of different advertising strategies on digital platforms and to assess the impact of exposure to these strategies on the probability of use initiation and increased consumption of conventional tobacco and new tobacco products among young Spaniards.

**METHODS:**

An online survey was conducted on a representative sample of 1730 young Spaniards aged 16–21 years in November 2022 using a comprehensive approach, considering all possible relevant factors and perspectives regarding the issue of the study. A descriptive analysis and two adjusted logistic regression models were employed to explore the association of exposure to digital platforms with the likelihood of conventional tobacco and new tobacco product use among this population.

**RESULTS:**

Among the participants, 83.2% reported witnessing individuals smoking, 61.6% observed identifiable logos or explicit advertisements, and 77.6% encountered indirect product placement on social media. Notably, exposure to conventional tobacco product placement (AOR=1.71; 95% CI: 1.27–2.30) emerged as the variable most significantly associated with an increased probability of tobacco use. Furthermore, exposure to advertising related to new tobacco products (AOR=2.47; 95% CI: 1.90–3.21) was linked to a heightened likelihood of subsequent use. Similarly, the direct promotion of these novel products is also associated with a higher probability of conventional tobacco use (AOR=1.58; 95% CI: 1.21–2.07).

**CONCLUSIONS:**

A reciprocal impact was identified, with the promotion of vaping being associated with an elevated probability of engaging in conventional tobacco smoking. Urgent attention is warranted for formulating public policies to mitigate the adverse effects of such insidious indirect advertising practices on digital platforms.

## INTRODUCTION

Throughout history, the tobacco industry has employed a diverse array of strategies to promote tobacco use among the youth demographic^1-3^. A pivotal tactic involves associating tobacco consumption with leisure and entertainment. Consequently, considerable research has been directed toward exploring the potential nexus between tobacco advertising, promotion, and film sponsorship and its impact on adolescents. Many studies have consistently demonstrated that heightened exposure to tobacco imagery in films and television constitutes a significant contributing factor to subsequent tobacco use among young individuals^4-9^.

Experimental investigations have further substantiated a causal relationship, revealing that exposure to tobacco imagery in films correlates with more favorable attitudes toward individuals who smoke^10^ and an increase in adolescents’ self-reported intentions to engage in smoking behavior^11^. These findings underscore the influential role of cinema and television depictions in shaping attitudes and behavioral intentions related to tobacco use among the youth.

While extensive research has probed into the impact of exposure to tobacco use in films on individual smoking behaviors, there needs to be further information regarding the influence of tobacco content on alternative communication platforms. A recent examination of tobacco content within Netflix and major cable television series revealed that a striking 86% of the programs incorporated tobacco imagery^12^. Similar exposure patterns were identified in studies conducted in the United Kingdom^13,14^. Although limited research pertains to tobacco imagery in series in Spain, available evidence indicates a notably high prevalence of exposure to tobacco and other substance use within domestically produced teen series and video-on-demand (VOD) platforms^15^.

The literature also sheds light on the frequency of tobacco product promotion on social media platforms^16-19^, with over half of adolescent participants reporting exposure to such content^20,21^. Moreover, additional research has established an association between social media exposure and e-cigarette use among young individuals^22-26^. These collective findings underscore the importance of extending our examination beyond conventional film media to comprehend the nuanced landscape of tobacco content and its potential impact across diverse communication platforms.

It is crucial to recognize that the realm of ‘digital smoke’ encompasses a multitude of exposure channels, including VOD and social media platforms, conventional television, and cinema, among others. Furthermore, the nature of promotional content has undergone a profound transformation, transitioning from the direct impact of logos and trademarks to more nuanced marketing strategies such as product placement or the subtle inclusion of individuals smoking. These subtle approaches are deliberately designed to navigate and circumvent state regulations.

The complexity of this landscape is further heightened by the introduction of novel forms of smoking, such as heated tobacco, waterpipes, and e-cigarettes, alongside conventional tobacco products. This amalgamation results in an ecosystem characterized by diverse stimuli, all strategically aimed at promoting the utilization of tobacco products and their derivatives, particularly among the youth demographic. Understanding this intricate interplay is imperative for developing comprehensive strategies to counteract the multifaceted influences within the evolving landscape of tobacco promotion.

A dual-purpose framework drives this study. Firstly, it compares the potential impact of the most commonly utilized digital platforms among adolescents and young adults – specifically, social media and VOD. The objective is to discern whether this impact is inclined towards conventional tobacco or extends to new tobacco products. Secondly, the study aims to delineate the nature of exposure, distinguishing between direct and indirect forms. Examples include identifiable brands and logos, the portrayal of someone actively smoking, or the indirect placement of a cigarette or tobacco product within audiovisual content. The main goal is to evaluate how these diverse forms of exposure may be associated with a higher likelihood of smoking conventional tobacco or using new tobacco products within the targeted population.

## METHODS

### Participants and sample

A survey was undertaken among individuals in Spain aged 16–21 years. Participants were selected through an online panel method designed to be representative of the target population in Spain. The data collection process finished in November 2022, resulting in a final sample size of 1730 interviews.

### Measures

An *ad hoc* questionnaire, provided in the Supplementary file, was developed following a comprehensive literature review of analogous research focusing on variables associated with the utilization and attitudes of young individuals towards both conventional tobacco and emerging tobacco products.

*Sociodemographic characteristics*

All participants were systematically queried regarding key demographic parameters, encompassing age, gender, urbanization level of residence (<10000; 10001–50000; 50001–500000; and >500000 inhabitants), employment status, their education level and their parents’ education level (below primary, primary, secondary, university), and self-perceived social class (lower class/lower-to-middle class; middle-to-upper class/upper class).

*Use and attitude towards tobacco and new tobacco products among young people*

This section presents information on the utilization and prevalence of conventional and new tobacco products. Participants were categorized as: ‘Individuals who have engaged in tobacco smoking in the last 30 days’ and ‘Individuals who have utilized new tobacco products in the last 30 days’, regardless of the frequency of use during this period. Furthermore, participants were probed about their attitudes and beliefs concerning the use of both tobacco and new tobacco products such as e-cigarettes and waterpipes, in addition to inquiring about the prevalence of usage among their family and friends.

*Leisure time and use of VOD and social media platforms among young people*

In this section, participants were asked about their leisure activities, specifically focusing on their engagement with social media and VOD platforms and the frequency of such engagement. Participants were prompted to recall instances, within the last 30 days, of encountering an identifiable logo or brand, observing someone smoking, or encountering any form of tobacco-related product in audiovisual content – be it associated with conventional tobacco or new tobacco products.

### Statistical analysis

A descriptive analysis of the study’s dependent and independent variables was conducted, employing frequencies and percentages. Furthermore, two binary logistic regression models were employed to ascertain the variables exerting the most significant influence on the likelihood of engaging in smoking tobacco or using new tobacco products (e-cigarettes and waterpipes) at least once in the last 30 days, from which adjusted odds ratios (AORs) and 95% confidence intervals (95% CI) were calculated. The selection criterion for the independent variables that were included in each model was theory-driven selection based on the existing literature on the subject. The models yielded Nagelkerke’s coefficients of determination, with R² values of 0.12 and 0.18 for the dependent variables of at least monthly use of conventional tobacco and at least monthly use of e-cigarettes or waterpipes, respectively. The predetermined significance level was set at <0.05 (two-tailed). Data analysis was performed using IBM SPSS Statistics version 27.

## RESULTS

### Use of conventional tobacco and new tobacco products among young people

Table 1 shows the distribution of variables regarding the use of tobacco and new tobacco products in young people aged 16–21 years. Over half of the respondents (57.8%) smoked tobacco at some point in their lives. The most commonly smoked tobacco among this group were cigarettes sold in packs (77.5%), followed by roll-your-own (hand-rolled) cigarettes (63.1%) and nicotine-free flavored vaping devices (43.7%). A total of 50.6% of the participants reported smoking some tobacco or using new tobacco products at least once in the last 30 days; 54.5% of male respondents reported smoking tobacco or using new tobacco products at least once in the last 30 days, while the percentage of women who reported doing so was 46.4%.

**Table 1:** Distribution of variables regarding the use of conventional tobacco and new tobacco products among young people aged 16–21 years, a cross-sectional survey, Spain, 2022 (N=1730)

*Variables*	*% (n)*
**Have you ever smoked tobacco?**	100 (1730)
Yes	57.8 (1001)
No	42.2 (729)
**What type of tobacco have you smoked?**	100 (1001)
Cigarettes sold in a pack	77.5 (775)
Roll-your-own cigarettes	63.1 (631)
Heated tobacco products such as IQOS	12.3 (123)
Pipe tobacco	5.8 (58)
Cigars	13.3 (133)
**Smokes tobacco at least monthly**	100 (1730)
Yes	33.2 (574)
No	66.8 (1156)
**Have you ever smoked e-cigarettes or waterpipes?**	100 (1730)
Yes	67.8 (1173)
No	32.2 (557)
**What type of e-cigarette or waterpipe have you used?**	100 (1173)
Nicotine-free flavored vapes, vape pods or e-liquids	64.4 (755)
Vape pods or e-liquids with nicotine	39.2 (460)
Nicotine-free waterpipes (shisha, hookah, narghile)	40.6 (476)
Waterpipe (shisha, hookah, narghile) with nicotine	35.0 (411)
**Use of e-cigarettes or waterpipes at least monthly**	100 (1730)
Yes	39.6 (686)
No	60.4 (1044)

### Beliefs and attitudes towards smoking and new tobacco products

Table 2 shows the main variables related to attitudes toward smoking and using new tobacco products; 48.8% of the respondents preferred to date people who did not smoke cigarettes (strongly agree or agree), while 71.6% did not mind if their friends smoked e-cigarettes or waterpipes when they went out. In addition, 39.2% said these products were present in their leisure time; 25.9% believed that the detrimental effects of conventional tobacco are ‘exaggerated’. This percentage increased to 36.2% when asked about the harmful effects of vaping and smoking waterpipes.

**Table 2:** Beliefs and attitudes towards smoking and use of new tobacco products among young people aged 16–21 years, a cross-sectional survey, Spain, 2022 (N=1730)

	*Strongly disagree %*	*Disagree %*	*Agree %*	*Strongly agree %*
I prefer going out with people who don’t smoke cigarettes or roll-your-own cigarettes	22.9	28.3	26.1	22.7
I don’t mind my friends vaping or smoking waterpipes when we go out	11.2	17.1	30.5	41.1
The detrimental effects of vaping and smoking waterpipes are exaggerated	28.6	35.1	25.9	10.4
The harmful effects of smoking cigarettes or roll-your-own cigarettes are exaggerated	43.8	30.3	16.6	9.3
E-cigarettes or waterpipes are present in how I spend most of my leisure time	33.6	27.1	27.9	11.3
Smoking cigarettes makes characters in TV shows and movies appear interesting	29.6	26.4	32.2	11.9
I think smoking cigarettes makes flirting or making friends easier	36.7	26.8	27.4	9.1
Vaping or smoking waterpipes is better because it doesn’t leave a residual smell (in your mouth, on your clothing, or in the environment)	22.3	24.6	37.0	16.0
It seems easy to quit vaping or smoking waterpipes	19.8	28.8	32.7	18.6
Vaping or smoking waterpipes is trendy because many famous people do it (actors, streamers, influencers, etc.)	16.3	26.5	37.8	19.4

Positive attitudes towards smoking cigarettes remained high when asked about associations between smoking cigarettes and looking interesting (44.1%) or making it easier to flirt or make friends (36.5%). Favorable attitudes increased when asked about using new tobacco products. Hence, 53.1% believe e-cigarettes and waterpipes were better because they did not leave a residual smell, 51.4% felt that it is easier to quit these habits, and 37.6% see this as an excellent way to share experiences with friends. Regarding the impact of social media, 57.2% of the participants considered vaping and smoking waterpipes trendy because famous people use these devices (such as actors, streamers, or influencers).

### Exposure to conventional tobacco and new tobacco products in digital spaces

In all, 67.0% of the respondents considered watching films or TV series as one of their main leisure activities at home, while 62.6% chose social media; 97.5% were regular users of at least one social media service, while 77.1% regularly used at least one VOD platform. Table 3 shows exposure to conventional and new tobacco products as a function of the digital space.

**Table 3:** Exposure to tobacco or new tobacco products as a function of the type of digital space among young people aged 16–21 years, a cross-sectional survey, Spain, 2022 (N=1730)

	*Has seen someone smoking*		*Has seen identifiable logos*		*Has seen product placement*		*Total*
	*Social media % (n)*	*VOD platforms % (n)*	*Social media % (n)*	*VOD platforms % (n)*	*Social media % (n)*	*VOD platforms % (n)*	
**Conventional tobacco**	61.4 (1061)	50.5 (873)	35.7 (617)	24.8 (429)	59.9 (1035)	44.1 (763)	82.5 (1427)
**New tobacco products**	70.1 (1213)	29.0 (502)	52.8 (913)	22.5 (389)	61.1 (1057)	27.7 (479)	83.0 (1436)
**Total**	83.2 (1440)	58.5 (1012)	61.6 (1066)	33.5 (579)	77.6 (1343)	51.8 (896)	91.3 (1579)

VOD: video-on-demand.

On social media, new tobacco products had the highest rates of exposure (seeing someone smoking, direct advertising related to tobacco use, and tobacco product placement). In general, social media far outweigh the audiovisual content of VOD platforms as a disseminator of conventional tobacco and new product content. However, conventional tobacco content had higher exposure on VOD platforms.

### Analysis of determining factors on conventional tobacco and its new forms of use

Table 4 shows the results of the two logistic regression models employed in the study. The dependent variables were: at least monthly use of conventional tobacco, and at least monthly use of e-cigarettes or waterpipes.

**Table 4:** Variables included in the logistic regression models, among young people aged 16–21 years, a cross-sectional survey, Spain, 2022 (N=1730)

*Variables*	*Use of conventional tobacco in the last 30 days ^a^*			*Use of vapes or waterpipes in the last 30 days ^b^*		
	*AOR ^c^*	*95% CI*	*p*	*AOR ^c^*	*95% CI*	*p*
Has seen someone smoking cigarettes	1.56	(1.16–2.10)	0.003	1.02	(0.77–1.36)	0.886
Has seen someone vaping or smoking waterpipes	0.79	(0.58–1.07)	0.131	1.37	(0.99–1.87)	0.051
Has seen product placement of conventional tobacco products	1.71	(1.27–2.30)	<0.001	1.56	(1.17–2.08)	0.002
Has seen product placement of vapes or waterpipes	1.20	(0.89–1.61)	0.233	1.62	(1.21–2.15)	0.001
Has seen tobacco-related direct advertising	1.23	(0.97–1.57)	0.093	0.90	(0.71–1.15)	0.393
Has seen direct advertising related to vaping or smoking waterpipes	1.58	(1.21–2.07)	0.001	2.47	(1.90–3.21)	<0.001
Gender (male)	1.29	(1.05–1.60)	0.017	1.42	(1.15–1.75)	0.001
Age	1.13	(1.06–1.21)	<0.001	1.17	(1.09–1.24)	<0.001
Has repeated an academic school year	1.75	(1.41–2.17)	<0.001	1.50	(1.21–1.87)	<0.001
Self-perceived social class (middle-to-upper class/upper class)	1.14	(0.92–1.41)	0.226	1.33	(1.08–1.64)	0.008
Constant	0.01		<0.001	0.01		<0.001

a Model 1 was adjusted for the following variables: has seen someone smoking, has seen someone vaping or smoking waterpipes, has seen product placement of conventional tobacco products, has seen product placement of vapes or waterpipes, has seen tobacco-related direct advertising, has seen direct advertising related to vaping or smoking waterpipes, gender, age, has repeated an academic school year and self-perceived social class.

b Model 2 was adjusted for the following variables: has seen someone smoking, has seen someone vaping or smoking waterpipes, has seen product placement of conventional tobacco products, has seen product placement of vapes or waterpipes, has seen tobacco-related direct advertising, has seen direct advertising related to vaping or smoking waterpipes, gender, age, has repeated an academic school year and self-perceived social class.

c AOR: adjusted odds ratio.

Indirect forms of exposure to smoking and new tobacco products (e-cigarettes and waterpipes) have a significant impact on both the dependent variables. In the model of the dependent variable, at least monthly use of conventional tobacco, respondents who had seen product placement of conventional tobacco products had higher odds of also being a smoker (AOR=1.71; 95% CI: 1.27–2.30). Those who had seen advertising related to vaping or smoking waterpipes were more likely to report themselves as current smokers (AOR=1.58; 95% CI: 1.21–2.07), as did youth exposed to direct advertising, such as having seen someone smoking cigarettes (AOR=1.56; 95% CI: 1.16–2.10). In the model of the dependent variable, at least monthly use of e-cigarettes or waterpipes, having seen advertising related to vaping or smoking waterpipes were more likely to be current waterpipe or e-cigarette users (AOR=2.47; 95% CI: 1.90–3.21), while having seen product placement of vaping or smoking waterpipes also showed significance (AOR=1.62; 95% CI: 1.21–2.15).

Regarding sociodemographic variables, the results showed that conventional tobacco smokers were more likely to be male (AOR=1.29; 95% CI: 1.05–1.60) and concurrent e-cigarette/waterpipe users (AOR=1.42; 95% CI: 1.15–1.75). In addition, as age increased, so did the likelihood of conventional tobacco use (AOR=1.13; 95% CI: 1.06–1.21) and, slightly more so, use of vaping or smoking waterpipes (AOR=1.16; 95% CI: 1.09–1.24). Repeating an academic school year also increased the odds of being a current smoker of tobacco or new tobacco products (AOR=1.75; 95% CI: 1.41–2.17 and AOR=1.50; 95% CI: 1.21–1.87, respectively).

## DISCUSSION

This study assessed the association between conventional tobacco and new tobacco product usage, exploring their association with the online media impact experienced by young individuals who are potential consumers of these products. As indicated by the results, there exists evidence of pervasive and normalized tobacco consumption among the youth, coupled with increasingly favorable attitudes toward new tobacco devices and derivatives.

Our findings underscore the enduring significance of conventional tobacco in shaping the smoking behavior of young individuals, with 33.2% reporting monthly tobacco smoking and 39.6% acknowledging monthly use of new tobacco products such as e-cigarettes and waterpipes. The elevated prevalence of unconventional tobacco product use among the youth aligns with outcomes observed in analogous studies^27-29^. Furthermore, our results reveal instances of dual use, where participants concurrently report the current use of two distinct tobacco products. This observation diverges from trends identified in other studies that suggest a shift towards substituting conventional cigarettes with e-cigarettes and other alternative tobacco products^30-32^.

The attitudes of young individuals toward conventional tobacco use and new tobacco product consumption exhibit notable distinctions. While enduring perceptions surrounding cigarettes persist, including beliefs that smoking aids in navigating complex social or psychological situations (looking interesting, and making it easier to flirt or make friends)^10,11^, our findings underscore an even more entrenched positivity toward new tobacco products. Consequently, many young individuals perceive these novel products as superior alternatives to smoking due to factors such as the absence of residual smells, ease of quitting, or their suitability for shared experiences with friends^16^.

This observation raises significant concerns, particularly in light of the escalating use of alternative tobacco products among the youth who, in contrast to conventional cigarettes, view this consumption more positively and less as a risk^20,24^. Consequently, it becomes imperative that comprehensive tobacco regulation and prevention strategies targeted at youth encompass all tobacco products, extending beyond conventional cigarettes or roll-your-own cigarettes.

Regarding exposure to tobacco industry promotional strategies, our findings revealed that the most overt form of exposure – identifiable logos or brands – was infrequent on social media and VOD platforms^8,9^. Given the robust legal measures implemented in Spain and neighboring countries, this outcome aligns with expectations. Nevertheless, nearly a third of respondents recalled encountering a logo or brand. In contrast, indirect advertising was pervasive in both digital domains, particularly on social media, which is subject to notably less regulation in Spain than content platforms.

More than half of the surveyed young individuals recollected observing someone smoking cigarettes on social media or VOD platforms, with exposure levels mirroring recall rates for indirect product placement. This level of exposure intensified when respondents were questioned about new tobacco products (e-cigarettes, waterpipes, and other), surpassing exposure to conventional tobacco on social media irrespective of the advertising strategy. Specifically, on social media, 70.1% of respondents remembered seeing someone smoking, 52.8% recalled identifiable logos or brands, and 61.1% recalled product placement. Exposure to new tobacco products decreased on VOD platforms; nonetheless, almost a third of the surveyed young individuals reported some form of exposure. Conventional tobacco maintains prevalence over new products on these content platforms.

In addition to attitudinal variables, the observation of someone smoking cigarettes or encountering cigarette product placement on digital media heightens the likelihood of young Spaniards self-reporting as current smokers. Interestingly, exposure to advertising or recognizable brands of vaping products also significantly increases the likelihood of engaging in conventional tobacco smoking, suggesting a potential ‘cross-contamination’ effect stemming from the influence of this form of advertising^25,26^.

Furthermore, our results underscore the substantial impact of exposure to new tobacco products on social media and VOD platforms, significantly influencing product use. Key predictors for usage encompass having witnessed someone vaping and encountering product placements for tobacco or e-cigarettes. Notably, the impact of identifiable logos and brands stands out, doubling the odds of being a current user of new tobacco products.

Regarding the sociodemographic profile of young consumers, being male increased the likelihood of using both conventional tobacco and vaping or waterpipes. Additionally, a positive association was observed between age and the likelihood of consuming these substances and products^30^.

### Strengths and limitations

One of the primary limitations inherent in this study is the susceptibility to participants’ response bias, recall bias, and social desirability bias. Additionally, given the study’s cross-sectional nature, the establishment of causality and temporality remains beyond the scope of inference. Moreover, there are some issues regarding the possibility of variables interfering with the logistic regression model that need to be addressed from a confirmatory rather than an exploratory approach.

Furthermore, even though the prevalence data on the use of conventional tobacco and new tobacco products are higher than the data from previous studies framed in Spain^33^, it is important to take into consideration that the operationalization of the concept of prevalence is not precisely the same, as explained in the Methods section. In addition, the target population of our research compared to the target populations of other previously published studies is not the same, which may definitely influence these different data.

Notwithstanding these limitations, our results show the tobacco industry’s adept utilization of popular digital platforms among young individuals, a phenomenon previously underscored by other studies in Spain^34^. This trend has facilitated the proliferation of covert advertising and practices that, while not explicitly illegal, remain outside the verbatim scope of legal norms governing tobacco advertising. These practices are deemed unethical and intentionally cultivate confusion, particularly among young individuals. To address this regulatory shortfall, The Madrid 2023 Declaration ‘For a Tobacco-Free Generation’ (a pivotal initiative unveiled at the European Conference on Tobacco or Health 2023) explicitly encourages the establishment of a suitable legislative framework^35^. This regulatory foundation should be designed to fortify the defences of children and adolescents against the perils of tobacco and nicotine products by strategically diminishing their attractiveness and economic accessibility, effectively reducing their appeal and affordability.

## CONCLUSIONS

This study represents an initial exploration into the prevalence of subtle and indirect advertising of tobacco and new tobacco products on digital platforms and social media, examining its potential impact on young individuals who constitute the primary consumer base of these platforms. Furthermore, it serves as a call to legislators for decisive action and the formulation of public policies that, grounded in the precautionary principle, comprehensively regulate the impact of such advertising on youth populations.

## Supplementary Material



## Data Availability

The data supporting this research are available from the authors on reasonable request.
