# Supplementary material for: The influence of social media and video-on-demand platforms on the appeal and consumption of tobacco and emerging tobacco products: A cross-sectional study
Source: Tob Induc Dis. 2024 Oct 25;22:10.18332/tid/194491. doi: 10.18332/tid/194491 (PMC11504195; doi:10.18332/tid/194491)
Supplement: Supplementary file 1 [file TID-22-171-s1.pdf]

Questionnaire: Use of tobacco and new tobacco products among youth in Spain

Introduction

This questionnaire focuses on health and leisure activities among young people living in Spain. The estimated duration of the survey is less than 10 minutes and all responses are anonymous.

Your collaboration and sincerity are very important.

Thank you in advance for your participation.

Characteristics of Smoking Tobacco

To get started, we'd like you to answer the following questions related to the use of traditional tobacco, including products such as cigarettes sold in packs, roll-your-own (hand rolled) cigarettes, IQOS, pipes and cigars.

Q.1 Have you ever smoked tobacco (cigarettes, IQOS, pipes, etc.)? *Do not consider it smoking if you only took a few puffs on someone else's cigarette (pack, roll-your-own, or IQOS, pipe, or cigar).*

- Yes 1
- No 2

(FILTER YES Q.1 = 1)

Q.2 What type of tobacco do you usually smoke?

- Cigarettes sold in packs 1
- Roll-your-own cigarettes 2
- Heated tobacco such as IQOS 3
- Pipe tobacco 4
- Cigars 5

(FILTER YES Q.1 = 1 / 2)

Q.3 How often have you smoked cigarettes in the last 30 days?

- Less than once a week 1
- Several times a week, but not daily 2
- Daily 3
- Never 4

(FILTER YES Q.3 = 3)

Q.4 How many cigarettes do you smoke a day, on average?

- ______________

Now we would like to ask about new tobacco product use, namely the use of e-cigarettes/vaping devices (vapes with or without flavors, with pods/capsules or liquids, with or without nicotine) and water pipes. pod

Q.5 <u>Have you ever used vaping devices (POD) or water pipes (shisha, hookah, narghile)?</u> *Do not consider it smoking if you only took a few puffs on someone else's vape or water pipe.* MULTIPLE ANSWERS.

1. Yes, **nicotine-free** flavored vapes, vape PODS/CAPSULES or e-liquids.
2. Yes, vape PODS/CAPSULES or e-liquids **with nicotine.**
3. Yes, **nicotine-free** water pipes (shisha, hookah, narghile).
4. Yes, water pipes (shisha, hookah, narguile) **with nicotine.**
5. No, I have never smoked vapes or water pipes.

(FILTER BASED ON Q.5)

Q.6 How often have you smoked vapes in the last 30 days?

- Less than once a week 1
- Several times a week, but not daily 2
- Daily or almost daily 3
- Never 4

(FILTER BASED ON Q.5)

Q.7 How often have you smoked water pipes in the last 30 days?

- Less than once a week 1
- Several times a week, but not daily 2
- Daily or almost daily 3
- Never 4

(FILTER BASED ON Q.5)

Q.8 Do you use or were you using e-cigarettes (vaping) as a substitute for tobacco, to reduce tobacco use, or to quit smoking?

- Yes, to quit smoking 1
- Yes, to reduce tobacco use 2
- No 3

(FILTER BASED ON Q.5)

Q.9 Where do you or did you usually smoke water pipes?

- At home 1
- At my friends’ or family’s homes 2

- On the street or at a park 3
- At a bar or water pipe café 4
- Other 5

Attitudes towards new tobacco products and alternative explanations.?
(EVERYONE RESPONDS)

Q.10 Below is a series of situations and behaviors that we have all experienced at some point. Within the context of the last 30 days, please respond to each question with one of the following: "no desire", "little desire", "strong desire" or "very strong desire",.

	No desire	Little desire	Strong desire	Very strong desire
a. (only for non-smokers Q1 = 2) to smoke a cigarette				
b. (only for non-smokers Q5 = 5) to vape				
c. (only for non-smokers Q5 = 5) to smoke a water pipe				

Q.11 To what extent do you agree or disagree with the following statements? [ROTATE]:

	STRONGLY AGREE	AGREE	DISAGREE	STRONGLY DISAGREE
a. I prefer going out with people who **DON'T SMOKE cigarettes or roll-your-own cigarettes.**				
b. I don't mind my friends **VAPING or smoking water pipes** when we go out.				
c. **The harmful effects of smoking cigarettes** or roll-your-own cigarettes are exaggerated.				
d. **The detrimental effects of vaping and smoking water pipes** are exaggerated.				

Q.12 Once again, please indicate to what extent you agree or disagree with the following statements. [ROTATE]:

	STRONGLY AGREE	AGREE	DISAGREE	STRONGLY DISAGREE
a. Smoking cigarettes makes characters on TV shows and movies look interesting.				

b. Smoking a cigarette helps a lot when someone is stressed or depressed.				
c. I think smoking cigarettes makes flirting or making friends easier.				
d. Vaping or smoking water pipes is better because it doesn't leave a residual smell (in your mouth, on your clothing, or in the environment).				
e. Vaping or smoking water pipes helps a lot when someone is stressed or depressed.				
f. It seems easy to quit vaping or smoking water pipes.				
g. Vaping or smoking water pipes is trendy because many famous people do it (actors, *streamers, influencers*, etc.).				
h. Vaping and smoking water pipes are good for sharing experiences with friends.				

Family and social environment and leisure time

(EVERYONE RESPONDS)

Now, we'd like to know a little more about your family and social environment.

Q.13 Please indicate if you have any close friends who smoke or use...MULTIPLE ANSWERS.

a. Cigarettes from a pack or roll-your-own
b. E-cigarettes or vaping devices
c. Water pipes, shishas, hookahs, narguiles

Q.14 Please indicate if someone in your household smokes or uses...MULTIPLE ANSWERS.

a. Cigarettes from a pack or roll-your-own
b. E-cigarettes or vaping devices
c. Water pipes, shishas, hookahs, narguiles

Q.15 Where do you usually go in your leisure time? MULTIPLE ANSWERS.

a. Shops or shopping centres.
b. Sports centres or athletic fields.
c. Parks or green spaces.
d. Friends’ houses.

e. Outside, on the street.

Q.16 When you're at home, which of the following activities do you usually do in your free time? MULTIPLE ANSWERS.

a. Watch films or series.
b. Read books, comic books, Manga…
c. Play videogames.
d. Play board games.
e. Lie on the sofa/on the bed.
f. Use social media.

Q.17 How much money do you spend weekly on going out with your friends, going to see a movie, having a bite to eat, etc.?

o Less than 5 €	1
o Between 5 € and 10 €	2
o Between 10 € and 15 €	3
o Between 15 € and 20 €	4
o Between 20 € and 30 €	5
o Between 30 € and 50 €	6
o Between 50 € and 100 €	7
o More than 100 €	8

Use of social media and digital platforms

The following questions are focused on understanding your habits regarding social media, digital platforms, and other types of communication media.

Q.18 How would you describe your activity on the social media and digital platforms listed below over the last six months?

	Frequent	Sporadic	Did not use it
a. Twitch			
b. Instagram			
c. Snapchat			
d. Facebook			
e. YouTube			
f. TikTok			
g. Twitter			
h. Other (indicate)			

Q.19 Approximately how often do you use the digital platforms and social media on which you are active?

- o Daily or almost daily 1
- o Several times a week 2
- o Several times a month 3
- o Less frequently 4

Q.20 When you use these digital spaces, how much time do you spend on them?

- o Less than 30 minutes 1
- o From 30 minutes to an hour 2
- o From one to two hours 3
- o From two to three hours 4
- o More than three hours 5

Q.21 How often did you watch traditional TV (DTT) during the last six months, regardless of whether it was on a television, a cell phone, a computer, a tablet, etc.?

- o Daily or almost daily 1
- o Several times a week 2
- o Several times a month 3
- o Less frequently 4
- o Never 5

Q.22 How would you describe your use of the following movie and series platforms during the last six months, regardless of whether it was on a television, a cell phone, a computer, a tablet, etc.?

	Frequent	Sporadic	Did not use it
a. Netflix			
b. Amazon Prime Video			
c. Disney+			
d. HBO Max			
e. Movistar+			
f. RTVE Play			
g. Atresplayer			
h. Mitele			
i. Other (indicate)			

Q.23 Approximately how often do you watch TV, movies and series on the digital platforms you use?

- o Daily or almost daily 1
- o Several times a week 2
- o Several times a month 3
- o Less frequently 4

Q.24 On average, how much time do you spend *per day* watching TV, films and series on these digital platforms?

- Less than 30 minutes 1
- From 30 minutes to an hour 2
- From one to two hours 3
- From two to three hours 4
- More than three hours 5

Q.25 Over the last six months, when using your usual digital platforms, social media, or watching TV, do you remember seeing someone…

Filter based on Q.18, Q.21 and Q.22 = FREQUENT	1. smoking cigarettes?	2.vaping or using water pipes?
a. Social Media		
b.		
c.		
d. Traditional TV (DTT)		
e. VOD (Video on Demand) Platforms		
f.		
g.		

Q.26. Over the last six months, when using digital platforms, social media, or watching TV, do you remember seeing product placement …

Filter based on Q.18, Q.21 and Q.22 = FREQUENT	1. product placement related to tobacco use (tobacco, lighters, rolling paper, cigars)	2. product placement related to vaping or smoking water pipes (vape devices, water pipes, refills)
a. Social Media		
b.		
c.		
d. Traditional TV (DTT)		
e. VOD (Video on Demand) Platforms		
f. ..		
g.		

Q.27 Over the last six months, when using digital platforms, social media, or watching TV, do you remember seeing advertising, promotions or logos associated with…

Filter based on Q.18, Q.21 and Q.22 = FREQUENT	1. traditional tobacco	2. vaping or smoking water pipes
a. Social Media		
b.		
c.		
d. Traditional TV (DTT)		

e. VOD (Video on Demand) Platforms		
f. ..		
g.		

DEMOGRAPHIC CHARACTERISTICS

QA. Gender:

QB. Age:

QC. Municipality: …………………… (to determine location, province and autonomous community)

QD. Check the option that best reflects your current status.

- I am a student 1
- I neither work nor study at the moment 2
- I work 3
- I work and study 4

QE. What is your level of education?

- **No formal education or unfinished Primary Education** 1
- **Primary Education (6th grade)** 2
- **1st cycle of Compulsory Secondary Education (ESO) (7th or 8th grade)** 3
- **2nd cycle of ESO (9th- 10th grade or Basic Vocational Education and Training)** 4
- **Secondary Education/High School Graduate (11th- 12th grade)** 5
- **Intermediate Vocational Education and Training** 6
- **Advanced Vocational Education and Training** 7
- **University Student or Degree** 8

QI. Have you ever repeated an academic school year?

- Yes, once 1
- Yes, two or more school years 2
- No 3

QE. On a different subject, and almost wrapping up, we would like to ask about your family environment. What is your mother's education level?

- **No formal education** or unfinished Primary Education 1
- **Primary Education** (6th grade, academic certificate, 6 years of schooling) 2
- **2nd cycle of ESO (**10th grade or 4 years of secondary education) 3
- **Secondary Education/High School Graduate** (BUP, COU) 4

- **Basic or Intermediate Vocational Education and Training** 5
- **Advanced and Certificate of Higher Vocational Education and Training** 6
- **University Degree, Master's Degree or Doctorate** 7
- I don´t know

8

QF. What is your father's education level?

- **No formal education** or unfinished Primary Education 1
- **Primary Education** (6th grade, academic certificate, 6 years of schooling) 2
- **2nd cycle of ESO (**10th grade or 4 years of secondary education) 3
- **Secondary Education/High School Graduate** 4
- **Basic or Intermediate Vocational Education and Training** 5
- **Advanced and Certificate of Higher Vocational Education and Training** 6
- **University Degree, Master's Degree or Doctorate** 7
- I don´t know 8

QG. What would you say is your family´s social class compared to other families in Spain?

- Upper class 1
- Upper/middle class 2
- Lower/middle class 3
- Lower class 4

© 2024 de Haro D. et al.
